# Feasibility and Safety of Autologous Dendritic Cell Vaccination Combined with Radio-Chemotherapy in Newly Diagnosed Glioblastoma: A Retrospective Single-Center Series

**DOI:** 10.3390/vaccines14020172

**Published:** 2026-02-12

**Authors:** Inés Esparragosa Vázquez, Ascensión López-Díaz de Cerio, Susana Inoges, Javier Aristu, Pablo Domínguez, Reyes García-Eulate, Marta Calvo-Imirizaldu, Javier Arbizu, María E. Rodríguez-Ruiz, Pablo Irimia, Marta M. Alonso, Felipe Prósper, Ricardo Díez-Valle, Jaime Gállego Pérez-Larraya

**Affiliations:** 1 Department of Neurology, Clínica Universidad de Navarra, Avenida Pío XII 36, 31008 Pamplona, Spain; iesparragos@alumni.unav.es (I.E.V.); pirimia@unav.es (P.I.); 2 Cell Therapy Unit, Clínica Universidad de Navarra, Avenida Pío XII 36, 31008 Pamplona, Spain; aslopez@unav.es (A.L.-D.d.C.); sinoges@unav.es (S.I.); fprosper@unav.es (F.P.); 3 Instituto de Investigación Sanitaria de Navarra (IdiSNA), C/Irunlarrea 3, 31008 Pamplona, Spain; mrruiz@unav.es (M.E.R.-R.); mmalonso@unav.es (M.M.A.); 4 Department of Radiation Oncology, Clínica Universidad de Navarra, Avenida Pío XII 36, 31008 Pamplona, Spain; jjaristu@unav.es; 5 Cancer Center, Clínica Universidad de Navarra, Avenida Pío XII 55, 31008 Pamplona, Spain; pdaniel@unav.es (P.D.); mgeulate@unav.es (R.G.-E.); mcalvoi@unav.es (M.C.-I.); jarbizu@unav.es (J.A.); 6 Department of Radiology, Clínica Universidad de Navarra, Avenida Pío XII 36, 31008 Pamplona, Spain; 7 Department of Nuclear Medicine, Clínica Universidad de Navarra, Avenida Pío XII 36, 31008 Pamplona, Spain; 8 Solid Tumors Program, Advanced Therapies for Pediatric Solid Tumors Group, Centro de Investigación Médica Aplicada (CIMA), Universidad de Navarra, Avenida Pío XII 55, 31008 Pamplona, Spain; 9 Department of Haematology and Haemotherapy, Clínica Universidad de Navarra, Avenida Pío XII 36, 31008 Pamplona, Spain; 10 Department of Neurosurgery, Hospital Universitario Fundación Jiménez Díaz, Avenida Avenida Reyes Católicos 2, 28040 Madrid, Spain; rdiezvalle@quironsalud.es

**Keywords:** glioblastoma, high-grade gliomas, dendritic cell vaccination, immunotherapy

## Abstract

Background: The prognosis of glioblastoma (GBM) patients remains poor. Dendritic cell (DC) vaccination has been investigated as an immunotherapy option, mainly in early-phase clinical studies. Herein, we report the feasibility, safety, and descriptive clinical and radiological outcomes of a retrospective series of newly diagnosed GBM patients treated with standard radio-chemotherapy and autologous DC vaccination as compassionate use. Methods: We retrospectively reviewed the medical and radiological records of patients with newly diagnosed GBM who received autologous tumor lysate–pulsed DC vaccination in addition to standard-of-care treatment at a tertiary academic center between 2009 and 2017. Clinical data, treatment characteristics, adverse events, survival outcomes, and radiological responses were collected and analyzed descriptively. Results: Twenty-four patients were included. All patients underwent surgical resection and were further treated with autologous tumor lysate–DC vaccination and standard radio-chemotherapy. Histology of GBM was confirmed in all patients. The first vaccine was administered in 75% of patients after a median of 21 days (range: 6–30 days) following surgery and prior to radiotherapy initiation. DC vaccination was continued following radiotherapy at specific time points, with no observed significant adverse events. Median OS was 21.1 months (95% CI, 27.9–75.0 months), and median PFS was 10.3 months (95% CI, 15.6–26.6 months). Presence of O6-methylguanine DNA methyltransferase (MGMT) promoter methylation was associated with longer survival and higher 12-month PFS rates, consistent with its established prognostic value. Radiological responses were retrospectively assessed according to RANO and RANO 2.0 criteria. Conclusions: In this retrospective single-center series, autologous DC vaccination administered as compassionate use in combination with standard radio-chemotherapy was feasible and safe in routine clinical practice. Survival and radiological outcomes are reported descriptively and should be interpreted with caution given the absence of a control cohort. These findings support further prospective controlled studies to properly assess the clinical role of DC vaccination in newly diagnosed GBM.

## 1. Introduction

Glioblastoma (GBM) is the most common malignant primary brain tumor in adults, accounting for approximately 45–50% of malignant primary central nervous system tumors [1]. Despite advances in surgical techniques and multimodal treatment strategies, the prognosis of patients with GBM remains dismal. The current standard of care consists of maximal surgical resection followed by concomitant radiotherapy and temozolomide, with adjuvant temozolomide, and more recently, the incorporation of tumor-treating fields (TTFs) [2,3,4]. Even with this optimal approach, median overall survival (OS) rarely exceeds 16–20 months, and virtually all patients experience tumor recurrence [2,4,5]. These outcomes highlight the urgent need for novel therapeutic strategies to improve long-term survival.

One of the major challenges in GBM treatment is its marked biological heterogeneity and profound resistance to conventional therapies. In addition, GBM is characterized by an immunosuppressive tumor microenvironment, including low tumor mutational burden, limited lymphocyte infiltration, dysfunctional antigen presentation, and the presence of immunosuppressive cell populations such as regulatory T cells and tumor-associated macrophages [6]. These features contribute to the classification of GBM as an immunologically “cold tumor” and have limited the success of immunotherapeutic approaches that have been effective in other solid malignancies.

In this context, a combination of cytotoxic chemotherapy and immunotherapy, so-called chemoimmunotherapy, has already been consolidated in the management of various solid tumors and is being actively investigated in clinical trials as a novel therapeutic paradigm for malignant glioma [7,8,9,10,11]. Multiple immunotherapeutic strategies are currently under investigation, including immune checkpoint inhibitors, chimeric antigen receptor (CAR) T-cell therapies, oncolytic viruses, and therapeutic cancer vaccines [12]. Among these, vaccination strategies aim to induce a durable and tumor-specific immune response and may be particularly appealing in the minimal residual disease setting after surgical resection.

Dendritic cell (DC) vaccination is among the most extensively studied vaccine-based immunotherapies in GBM. DCs are professional antigen-presenting cells that play a central role in orchestrating both innate and adaptive immune responses, particularly by activating tumor-specific T lymphocytes [13,14]. In DC vaccination, autologous DCs are generated ex vivo and loaded with tumor-associated antigens—commonly derived from autologous tumor lysate—before being administered back to the patient. This approach allows for the presentation of a broad repertoire of patient-specific tumor antigens, potentially overcoming tumor heterogeneity and promoting immunological memory.

Over the past two decades, multiple early-phase clinical trials have demonstrated that DC vaccination in GBM is feasible and generally well tolerated, with signals of immunogenicity and potential survival benefit in selected patient populations [15,16]. However, results across studies have been heterogeneous, and the clinical efficacy of DC vaccination has not yet been definitively established. Furthermore, assessing treatment response remains challenging, as immunotherapy may induce atypical radiological patterns, including pseudoprogression, complicating response evaluation using conventional imaging criteria.

Herein, we retrospectively describe the clinical and radiological outcomes of a series of patients with newly diagnosed GBM treated with autologous DC vaccination as compassionate use in addition to standard radio-chemotherapy. This study was not designed to assess comparative efficacy between treatment paradigms, but to describe feasibility, safety, and descriptive clinical outcomes in a real-world setting.

## 2. Materials and Methods

### 2.1. Patients

We retrospectively reviewed all adult patients with newly diagnosed GBM treated at our institution who underwent complete or near-complete resection, followed by standard radiotherapy with concomitant and adjuvant temozolomide, and who additionally received vaccination with autologous DC pulsed with tumor lysate as compassionate use. An attempt at maximum resection with a residual tumor volume less than 1 cc was mandatory for receiving the vaccination treatment.

A neuropathologist confirmed histological diagnosis in accordance with the 2016 World Health Organization (WHO) classification. Eligible patients should not have received any prior antitumor treatment. A comprehensive retrospective chart review of medical records and magnetic resonance imaging (MRI) scans was performed to ascertain key variables. Data collected included age at diagnosis, gender, presenting symptoms, perioperative neurological status, and Karnofsky Performance Status (KPS), use of corticosteroids, extent of surgery, histopathological diagnosis, IDH1/2 gene mutations, and MGMT promoter methylation status, radiation and chemotherapy dosages, response assessment, time to tumor progression, and date of death or last follow-up.

MRI scans, including T2/fluid-attenuated inversion recovery (T2/FLAIR) and pre- and post-gadolinium T1-weighted images (T1WIs), were reviewed at key time points: post-operative (within 72 h), before radiotherapy, 1 month post-radiotherapy, and every 2 months thereafter. Response was assessed according to the Response Assessment in Neuro-Oncology (RANO) criteria and RANO 2.0 criteria [17,18]. Patients with fewer than two postoperative MRI scans were excluded from the final radiological analysis. Written informed consent was obtained from all patients before treatment, and the study received Institutional Review Board approval at our institution (register number CEI-UN 2020.102, 17 September 2020).

### 2.2. Dendritic Cell Vaccine Production

All steps of vaccine production (dendritic cell generation, antigen loading, and maturation) were carried out at the Cell Therapy Laboratory in Pamplona, Navarra, Spain, under Good Manufacturing Practice conditions, as previously described in detail [19], ensuring batch-to-batch consistency and reproducibility.

Briefly, after receiving a fresh tumor sample from the operating room, tumor single-cell suspensions were obtained by mechanical disaggregation (GentleMACS™ Dissociator, Miltenyi Biotec, Bergisch Gladbach, Germany) and immediately frozen and stored. For tumor lysate, cells were thawed and subjected to 4 cycles of freezing and thawing, and the centrifuge supernatant was then filtered and irradiated at 54 Gy and stored at −20 °C until use.

Peripheral blood mononuclear cells were obtained by leukapheresis within two weeks after surgery and at least seven days after dexamethasone tapering. CD14^+^ monocytes were isolated by immunomagnetic separation (CliniMACS™, Miltenyi Biotec, Bergisch Gladbach, Germany) and cultured at a density of 2 × 10^6^ cells/mL in AIM-V medium (Gibco, Grand Island, NY, USA), supplemented with antibiotics, interleukin-4 (IL-4; 1000 IU/mL; R&D Systems, Minneapolis, MN, USA), and granulocyte–macrophage colony-stimulating factor (GM-CSF; 1000 IU/mL; Leukine™, Genzyme Corporation, Cambridge, MA, USA), using culture bags (CellGenix, Freiburg, Germany). On day 4, additional IL-4 (500 IU/mL) and GM-CSF (500 IU/mL) were added, and cells were harvested on day 7.

Such immature DCs were adjusted at 10^7^ cells/mL and pulsed with autologous tumor lysate for 2 h at 37 °C and 5% CO^2^. To induce DC maturation, 50 ng/mL of tumor necrosis factor (TNF)-α (Beromun™, Boehringer Ingelheim, Ingelheim, Germany), 1000 UI/mL of interferon (IFN)-α (Intron A™, Schering Corporation, Berlin-Wedding, Germany) and 20 ng/mL of Poly I:C (Amersham, Little Chalfont Buckinghamshire, UK) were added to the medium, and cells were placed in culture bags at 2 × 10^6^ cells/mL. Mature DCs were harvested on day 8 and cryopreserved in individual aliquots until administration. An intended dose of 1 × 10^7^ DC was used for each vaccine administration, although lower cell numbers were obtained in some cases. Cell viability was assessed prior to each administration as part of routine quality control procedures.

Importantly, the vaccine production process was identical to that used in our previously published phase II clinical trial (NCT01006044), in which the manufacturing protocol is described in full technical detail [8].

Overall, the DC vaccine was fully autologous, being generated from patient-derived peripheral blood monocytes and pulsed with autologous tumor lysate obtained from the corresponding surgical tumor specimen.

### 2.3. Treatment Schedule

Standard external beam radiation therapy and concomitant chemotherapy with temozolomide 75 mg/m^2^/day was initiated 4 weeks following maximal safe resection, followed by up to 6–12 cycles of adjuvant treatment with temozolomide 150–200 mg/m^2^/day over 5 consecutive days every 28 days or until tumor progression.

DC vaccines were administered intradermally. The first administration was scheduled one week prior to the start of radiation therapy, and the second one 3 weeks after radiotherapy completion. Thereafter, the following two vaccines were administered monthly, the next four bi-monthly, and subsequent ones quarterly until the end of all available doses. During adjuvant treatment with temozolomide, DC vaccines were infused on day 21 of the corresponding cycle to benefit from the theoretical recovery from leukopenia.

When tumor progression occurred, patients were treated at the physician’s discretion. When still available, the possibility of maintaining vaccine administration along with second-line therapy was offered.

### 2.4. Statistical Analysis

Data for continuous variables are presented as medians with ranges, and for categorical variables as absolute numbers and percentages. OS was calculated as the interval of time from surgery to the date of death or last follow-up. Progression-free survival (PFS) was defined as the time from surgery to the date of progression, as established by the RANO criteria [17] and confirmed by RANO 2.0 criteria [18] in the post hoc retrospective analyses. Both OS and PFS were estimated using the Kaplan–Meier method with a 95% confidence interval [95% CI] calculated using the Greenwood method. The log-rank test was used to compare OS and PFS according to MGMT promoter methylation status, and the results were validated with the Tarone–Ware test. Prognostic factors for survival times were examined by a stepwise Cox regression model. Factors included in the model were age, sex, extent of surgery, KPS, number of vaccines received, IDH mutation, and MGMT promoter methylation. All of the analyses were performed using statistical software R version 4.1.0 (R Core Team 2020) and SPSS v24.0.

## 3. Results

### 3.1. Patient Characteristics

Between January 2009 and May 2017, twenty-four Spanish patients (13 women [54%] and 11 men [46%]) with newly diagnosed GBM were treated at the Clinica Universidad de Navarra, a single tertiary referral center, with autologous tumor lysate–DC vaccination as compassionate use in addition to surgical resection and standard radio-chemotherapy. Median age at the beginning of treatment was 61 years (range: 43–78 years), and median pre-surgical KPS was 80% (range: 40–100%). The most frequent presenting symptoms were hemiparesis or hemiplegia in 10/24 patients (42%), headache in 9/24 patients (37.5%), cognitive behavior in 6/24 patients (25%), aphasia in 5/24 patients (21%), visual alteration in 4/24 patients (17%), and seizures in 4/24 patients (17%).

All patients underwent 5-Aminolevulinic Acid-guided surgery, and an attempt at complete or near-complete resection of the contrast-enhancing tumor was performed in every case based on preoperative MRI. The former was achieved in 20 out of the 24 patients (83%). In the remaining 4 patients (17%), early postoperative MRI showed subtotal resection with a residual tumoral contrast enhancement volume less than 1 cc.

Histologic diagnosis of GBM was confirmed in all patients. The molecular analysis revealed IDH1 mutation in 1/24 patients (4%), whereas the remaining (23/24 patients, 96%) were IDH1 and IDH2 wild type. Thirteen out of 24 patients (54%) had an EGFR alteration. The MGMT promoter was methylated in 13/24 patients (54%) and unmethylated in 11/24 patients (46%).

The patients’ baseline characteristics are summarized in Table 1.

### 3.2. Treatment

Median post-surgical KPS was 70% (range: 40–100%). Following surgery, all patients were treated with intensity-modulated radiation therapy (IMRT). Median time since tumor resection to radiotherapy was 29 days (range: 19–49 days), and median administered dose was 70 Gy (range: 50–72.5 Gy). Concomitant chemotherapy with temozolomide was administered in all cases, with adequate tolerance and no need for dose reduction or discontinuation during this period. Four weeks after simultaneous chemo-radiotherapy completion, patients started treatment with adjuvant temozolomide at a dose of 150–200 mg/m^2^ per cycle. A median of 6 cycles per patient (range: 3–12 cycles) was administered. Adjuvant chemotherapy was globally well tolerated, with hematologic grade 1 or 2 toxicities occurring in 5/24 patients: grade 2 neutropenia in 3/24 patients (13%) and grade 1–2 thrombocytopenia in 2/24 patients (8%). Non-hematologic adverse events were mild and consisted of cutaneous reaction in 1/24 patients (4%) and nausea or vomiting in 3/24 patients (13%). Treatment-related toxicity did not lead to permanent cessation of chemotherapy. After tumor progression, 5/24 patients were re-operated (21%), 2/24 patients were re-irradiated (8%), and 18/24 (75%) received subsequent chemotherapy: 6/24 patients received bevacizumab alone (25%); 4/24 patients received irinotecan plus bevacizumab (17%); and 8/24 patients received temozolomide alone (33%).

Concerning DC vaccines, enough tumor lysate and DC were available in every case to produce at least 2 vaccination doses (median 7; range: 2–17). In all cases except 1 patient, steroid tapering could be performed within a few days after surgery, and the first vaccine was administered with a median time after surgery of 21 days (range: 6–30 days) in 18/24 patients (75%). In the remaining 6/24 patients (25%), the first vaccine administration was delayed after radiotherapy initiation. The median number of administered vaccines was 6 (range: 2–16 vaccines). No injection site reactions and no other adverse events attributable to vaccination were observed.

### 3.3. Descriptive Survival and Radiological Outcomes

Given the retrospective, uncontrolled nature of the study, all survival and radiological outcomes are presented for descriptive purposes only and do not allow conclusions regarding treatment efficacy.

All patients were dead at the time of the current analysis. Tumor progression was the primary cause of death in all 24 patients. DC vaccination was discontinued in all patients based on patient and investigator’s decision at the moment of progression.

The median OS was 21.2 months (95% CI, 27.9–75.0 months). Observed survival times were centered around this median value; higher values reflect the upper bounds of wide confidence intervals and do not correspond to individual observed survival durations. The 12-month and 24-month OS rates were 69.7% (95% CI, 49.2% to 83.2%) and 39.2% (95% CI, 20.7% to 57.7%), respectively. The median PFS was 10.3 months (95% CI, 15.6–26.6 months). The 12-month and 24-month PFS rates were 48.5% (95% CI, 26.8% to 67.6%) and 12.1% (95% CI, 2.5% to 31.2%), respectively.

**Figure 1:**
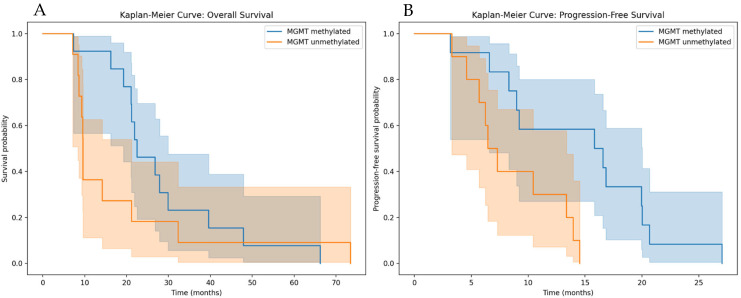
Overall survival (OS) and progression-free survival (PFS) curves according to MGMTp methylation status (**A**,**B**). PFS and OS in the whole series are shown with their corresponding 95% confidence interval (represented by the shaded areas in the curves). The presence of a MGMTp methylation was associated with a trend towards a longer median OS and PFS (*p* < 0.05).

Median OS among patients with MGMT methylation was 26.8 months (95% CI, 39.6–66.3 months) vs. 9.6 months (95% CI, 21.2–75.0 months) among those without methylation (*p* = 0.03; Figure 1A). The presence of MGMT methylation was associated with longer OS and PFS, consistent with its established prognostic role in glioblastoma, with a higher 12-month OS rate (100% vs. 36.4%, *p* = 0.008). Regarding PFS, patients with MGMT methylated promoter had longer PFS (16.3 months; 95% CI, 11.4–26.6 months) than those without methylation (6.4 months; 95% CI, 13.2–14.3 months; *p* = 0.03; Figure 1B). Presence of MGMT methylation was also associated with a higher rate of 12-month PFS rate (64.2% vs. 30%, *p* = 0.007). MGMT promoter methylation was analyzed as a descriptive prognostic variable, given its established role in glioblastoma, and not as a marker of treatment effect.

Imaging-based tumor response evaluation was performed on all patients following RANO and RANO 2.0 criteria. Radiological response assessment was performed retrospectively and should be interpreted with caution, particularly in the context of immunotherapy-related imaging changes.

Four patients were excluded from the imaging analysis due to a lack of radiological follow-up or an absence of imaging records in our digital system for RANO criteria analyses. Confirmed objective responses (i.e., complete response [CR], partial response [PR], or stable disease [SD]) were observed in 11/20 patients, resulting in a response rate of 55%. The best response was CR in 3 patients (15%), whereas 1/20 patients (5%) achieved PR and 7/20 patients (35%) SD. Nine patients (45%) had progressive disease as the best response to treatment.

According to the RANO 2.0 criteria, five patients were excluded from the imaging analysis due to a lack of sufficient post-radiotherapy images to perform the analysis. Confirmed objective responses were observed in 14/19 patients, resulting in a response rate of 74%. The best response was PR in 1 patient (5%), whereas 13/19 patients (68%) achieved SD. Five patients (26%) had progressive disease as the best response to treatment.

The median duration of response in patients with an objective response or stable disease was 5.6 months (95% CI [7.1–21.1 months]) according to the RANO criteria, and 3.3 months (95% CI [9.4–25.6 months]) for RANO 2.0 criteria.

## 4. Discussion

In this small, retrospective, single-center series of newly diagnosed GBM patients, DC vaccination following tumor resection, in addition to standard radio-chemotherapy, was feasible in real-world clinical practice. Aside from the expected toxicities associated with radio-chemotherapy, DC vaccination was safe and did not induce any adverse events. Furthermore, survival times and objective responses were within the range reported in selected series, but must be interpreted with caution given the study design.

Previous evidence from early-phase (phase I–II) clinical trials has reported heterogeneous survival outcomes in selected patient populations associated with dendritic cell vaccination in patients with GBM compared with matched or historical control cohorts, with reported median OS ranging from approximately 15 to 40 months in newly diagnosed patients [7,8]. Only a limited number of these studies—including one phase III trial—are randomized investigations designed to provide a higher level of evidence regarding efficacy [15,20]. However, methodological limitations of these studies preclude drawing firm conclusions, and have not yet been able to rigorously demonstrate the true benefit of this therapeutic approach.

As it is well established and consistently demonstrated with current standard radio-chemotherapy, and in accordance with previous series of GBM patients additionally treated with DC vaccination, MGMT-methylated patients exhibit higher response rates and longer survival times. In the present series, the median OS among patients with MGMT methylation was 26.8 months (95% CI, 39.6–66.3 months), compared with 9.6 months (95% CI, 21.2–75.0 months) among those without methylation [2]. As previously reported by Liau et al., MGMT methylation remains a strong prognostic factor in patients receiving standard chemoradiotherapy, including those treated with dendritic cell vaccination [15].

Currently, the heterogeneity across cohort series and in trial designs—including differences in DC vaccine production, antigen sources, and administration schedules—makes direct comparisons highly challenging [21]. In previously reported studies of newly diagnosed GBM, DC vaccination was generally administered in conjunction with standard radiotherapy and chemotherapy, similar to the regimen used in the present work. However, defining the optimal DC dose remains challenging, as numerous factors—such as the DC subtype, vaccination schedule, and route of administration, among others—may influence the anti-tumor immune response and overall efficacy [7]. Importantly, in this study, we demonstrated that DC vaccine production can be successfully completed, and the first dose can be administered shortly after tumor resection, before the initiation of radio-chemotherapy, approximately four weeks following surgery.

In an exploratory subgroup analysis, we analyzed whether patients who received dendritic cell vaccination after radiotherapy showed survival outcomes comparable to those reported in the overall cohort, with no difference in OS nor PFS results between groups. However, the small number of patients precludes any definitive conclusions regarding the impact of vaccination timing on survival.

Other well-known factors that have influenced previously reported outcomes include the extent of resection and patient selection [10,22,23,24,25]. In most of the previously published studies, the enrollment was restricted to patients who had undergone extensive surgical resection, resulting in minimal postoperative residual disease, in order to enhance the efficacy of the vaccine-induced antitumor immune response. However, in some series, patients who had undergone only a biopsy or no surgical resection at all were also included. In our cohort, complete tumor resection, as achieved, may have contributed to the observed survival outcomes, independently of the effect of vaccination, with a median OS of 21.2 months (95% CI, 27.9–75.0 months). The absence of significant residual tumor volume could be particularly relevant, as it not only reduces tumor burden but may also modulate the local immunosuppressive microenvironment, thereby facilitating vaccine efficacy.

Regarding inclusion criteria, patients aged over 70 years and those with a post-surgical KPS below 70% are typically excluded from most clinical trials. In the present study, however, enrollment was permitted for patients up to 78 years of age and for those with a postoperative KPS as low as 40%. Although these subgroups are often excluded from conventional clinical trials, their inclusion did not appear to have a significant impact on OS in this series, nor did it increase the incidence of treatment-related adverse events compared with previously published series [8,15,25,26,27,28]. This supports the feasibility of DC vaccination in a broader patient population than that typically enrolled in clinical trials.

Regarding tolerability, DC vaccination for GBM has demonstrated an excellent safety and tolerability profile across clinical trials. The most frequently observed side effects related to DC vaccination are mild, transient local skin reactions at the injection site, such as itching, erythema, and pain [7,29]. In our small series, DC vaccination was likewise well tolerated. No injection-site reactions or vaccination-related adverse events were documented during the study period. The most common adverse events observed were grade 1–2 cytopenia related to radio-chemotherapy, occurring in 21% of patients, and mild gastrointestinal symptoms in 13%, both comparable to rates reported in the literature [2,30].

The present study has several limitations that should be acknowledged. First, its retrospective design and small sample size (*n* = 24) limit the ability to draw definitive conclusions regarding the efficacy of DC vaccination. Importantly, the present study was not designed to assess clinical efficacy, and no causal inference regarding the impact of DC vaccination on survival can be made. Furthermore, no comparative cohort or quality-of-life assessment was available due to the retrospective nature of the study. Second, the absence of immune response analysis and correlative studies makes it difficult to determine whether patient outcomes are directly attributable to vaccine-induced immune effects, thereby precluding firm conclusions about the impact of this additional therapy. Third, patients included in this study were treated between 2009 and 2017, when histological diagnoses were based on the 2016 WHO classification. Accordingly, the sole patient initially classified as an IDH-mutated GBM would now be categorized as a grade 4 IDH-mutant astrocytoma under the 2021 WHO classification, and would be excluded from the analyses. This may have contributed to a slight improvement in the OS outcomes observed in this series.

At the time when the study was conducted, the RANO criteria were considered the standard for radiological response assessment. Consequently, progression may have been identified earlier than with the currently adapted RANO 2.0 criteria, as they include T2/FLAIR in the measurable disease assessment and use the post-surgical MRI as the baseline (rather than the post-radiotherapy scan), potentially leading to the initiation of second-line treatments at an earlier stage.

To evaluate this, we performed a post hoc analysis comparing both radiological criteria, which showed a higher response rate according to RANO 2.0 (74% vs. 55%). In this context, treatment-related changes due to immunotherapy, which can mimic tumor progression on MRI, may have been misclassified as true progression under the RANO criteria, potentially leading to underestimation of actual responses [3,17,18].

Our findings highlight critical questions for future research in three key areas. First, the optimization of treatment protocols, including timing, duration, and the potential continuation beyond progression. Second, the exploration of combination strategies to enhance vaccine efficacy, particularly in conjunction with emerging immunotherapeutic approaches. Third, the identification of reliable biomarkers for patient selection and response prediction beyond MGMT promoter methylation. Several ongoing clinical trials are addressing these questions, investigating DC vaccination in combination with temozolomide (NCT04801147, NCT02649582, NCT03548571, NCT03395587, NCT04523688) or with immune checkpoint inhibitors (NCT04201873), based on promising preclinical evidence of immune modulation.

## 5. Conclusions

In this retrospective single-center series, autologous DC vaccination administered as compassionate use in combination with standard radio-chemotherapy was feasible and safe in patients with newly diagnosed glioblastoma in routine clinical practice. No vaccine-related serious adverse events were observed, and vaccine production and administration were successfully integrated into standard treatment workflows.

Survival and radiological outcomes are reported descriptively and must be interpreted with caution, given the retrospective design, limited sample size, and absence of a control cohort. Consequently, no conclusions regarding clinical efficacy or therapeutic benefit of dendritic cell vaccination can be drawn from this study.

These findings support the feasibility of this approach and provide real-world data that may inform the design of future prospective, controlled clinical trials aimed at properly evaluating the clinical role of dendritic cell vaccination in newly diagnosed glioblastoma.

## Figures and Tables

**Table 1:** Demographic and clinical features of the study population (*n* = 24).

Characteristic	Value
Sex, *n* (%)	
Male	11 (46)
Female	13 (54)
Age, years	
Median (range)	61 (43–78)
KPS at baseline, *n* (%)	
40	1 (4)
50	2 (8)
60	3 (13)
70	5 (21)
80	4 (17)
90	8 (33)
100	1 (4)
Clinical presentation, *n* (%)	
Paresis	10 (42)
Cognitive dysfunction	6 (25)
Seizures	4 (17)
Headache	9 (37.5)
Aphasia	5 (21)
Extent of surgery, *n* (%)	
Gross total resection	20 (83)
Subtotal resection	4 (17)
IDH1/2 mutation status, *n* (%)	
Mutated	1 (4)
Wild type	23 (96)
MGMT promoter methylation status, *n* (%)	
Yes	13 (54)
No	11 (46)
Time from surgery to DC vaccination, days	
Median (range)	23 (6–751)

Baseline demographic, clinical, and molecular characteristics of the study population. Data are presented as number (%) unless otherwise indicated. Age and time from surgery to DC vaccination are presented as median (range). Abbreviations: KPS, Karnofsky Performance Status; DC, dendritic cell; IDH, isocitrate dehydrogenase 1 or 2; MGMT, O6-methylguanine-DNA methyltransferase.

## Data Availability

The original contributions presented in this study are included in the article. Further inquiries can be directed to the corresponding author.

## Abbreviations

The following abbreviations are used in this manuscript:

GBM	Glioblastoma
DC	Dendritic cell
TTFs	Tumor-treating fields
OS	Overall survival
WHO	World Health Organization
MRI	Magnetic resonance imaging
KPS	Karnofsky Performance Status
MGMT	O6-methylguanine DNA methyltransferase
T2/FLAIR	T2/fluid-attenuated inversion recovery
T1W1	T1-weighted images
PFS	Progression-free survival
RANO	Response Assessment in Neuro-Oncology
IDH 1/2	Isocitrate dehydrogenase 1 or 2
CR	Complete response
PR	Partial response
SD	Stable disease
PD	Progressive disease
IMRT	Intensity-modulated radiation therapy

## References

[1] Ostrom Q.T., Price M., Neff C., Cioffi G., Waite K.A., Kruchko C., Barnholtz-Sloan J.S. (2023). CBTRUS Statistical Report: Primary Brain and Other Central Nervous System Tumors Diagnosed in the United States in 2016—2020. Neuro-Oncology.

[2] Stupp R., Mason W.P., Van Den Bent M.J., Weller M., Fisher B., Taphoorn M.J.B., Belanger K., Brandes A.A., Marosi C., Bogdahn U. (2005). Radiotherapy plus Concomitant and Adjuvant Temozolomide for Glioblastoma. N. Engl. J. Med..

[3] Youssef G., Rahman R., Bay C., Wang W., Lim-Fat M.J., Arnaout O., Bi W.L., Cagney D.N., Chang Y.-S., Cloughesy T.F. (2023). Evaluation of Standard Response Assessment in Neuro-Oncology, Modified Response Assessment in Neuro-Oncology, and Immunotherapy Response Assessment in Neuro-Oncology in Newly Diagnosed and Recurrent Glioblastoma. JCO.

[4] Stupp R., Taillibert S., Kanner A., Read W., Steinberg D.M., Lhermitte B., Toms S., Idbaih A., Ahluwalia M.S., Fink K. (2017). Effect of Tumor-Treating Fields Plus Maintenance Temozolomide vs Maintenance Temozolomide Alone on Survival in Patients With Glioblastoma: A Randomized Clinical Trial. JAMA.

[5] Hottinger A.F., Pacheco P., Stupp R. (2016). Tumor Treating Fields: A Novel Treatment Modality and Its Use in Brain Tumors. Neuro Oncol..

[6] Quail D.F., Joyce J.A. (2017). The Microenvironmental Landscape of Brain Tumors. Cancer Cell.

[7] Datsi A., Sorg R.V. (2021). Dendritic Cell Vaccination of Glioblastoma: Road to Success or Dead End. Front. Immunol..

[8] Inogés S., Tejada S., De Cerio A.L.-D., Gállego Pérez-Larraya J., Espinós J., Idoate M.A., Domínguez P.D., De Eulate R.G., Aristu J., Bendandi M. (2017). A Phase II Trial of Autologous Dendritic Cell Vaccination and Radiochemotherapy Following Fluorescence-Guided Surgery in Newly Diagnosed Glioblastoma Patients. J. Transl. Med..

[9] Huang B., Li X., Li Y., Zhang J., Zong Z., Zhang H. (2021). Current Immunotherapies for Glioblastoma Multiforme. Front. Immunol..

[10] Buchroithner J., Erhart F., Pichler J., Widhalm G., Preusser M., Stockhammer G., Nowosielski M., Iglseder S., Freyschlag C.F., Oberndorfer S. (2018). Audencel Immunotherapy Based on Dendritic Cells Has No Effect on Overall and Progression-Free Survival in Newly Diagnosed Glioblastoma: A Phase II Randomized Trial. Cancers.

[11] Schaller T.H., Sampson J.H. (2017). Advances and Challenges: Dendritic Cell Vaccination Strategies for Glioblastoma. Expert Rev. Vaccines.

[12] Majc B., Novak M., Kopitar-Jerala N., Jewett A., Breznik B. (2021). Immunotherapy of Glioblastoma: Current Strategies and Challenges in Tumor Model Development. Cells.

[13] Palucka K., Banchereau J. (2013). Dendritic Cell-Based Cancer Therapeutic Vaccines. Immunity.

[14] Sferruzza G., Consoli S., Dono F., Evangelista G., Giugno A., Pronello E., Rollo E., Romozzi M., Rossi L., Pensato U. (2024). A Systematic Review of Immunotherapy in High-Grade Glioma: Learning from the Past to Shape Future Perspectives. Neurol. Sci..

[15] Liau L.M., Ashkan K., Brem S., Campian J.L., Trusheim J.E., Iwamoto F.M., Tran D.D., Ansstas G., Cobbs C.S., Heth J.A. (2023). Association of Autologous Tumor Lysate-Loaded Dendritic Cell Vaccination With Extension of Survival Among Patients with Newly Diagnosed and Recurrent Glioblastoma: A Phase 3 Prospective Externally Controlled Cohort Trial. JAMA Oncol..

[16] Wheeler C.J., Black K.L., Liu G., Mazer M., Zhang X., Pepkowitz S., Goldfinger D., Ng H., Irvin D., Yu J.S. (2008). Vaccination Elicits Correlated Immune and Clinical Responses in Glioblastoma Multiforme Patients. Cancer Res..

[17] Wen P.Y., Macdonald D.R., Reardon D.A., Cloughesy T.F., Sorensen A.G., Galanis E., DeGroot J., Wick W., Gilbert M.R., Lassman A.B. (2010). Updated Response Assessment Criteria for High-Grade Gliomas: Response Assessment in Neuro-Oncology Working Group. JCO.

[18] Wen P.Y., van den Bent M., Youssef G., Cloughesy T.F., Ellingson B.M., Weller M., Galanis E., Barboriak D.P., de Groot J., Gilbert M.R. (2023). RANO 2.0: Update to the Response Assessment in Neuro-Oncology Criteria for High- and Low-Grade Gliomas in Adults. J. Clin. Oncol..

[19] Valle R.D., de Cerio A.L.-D., Inoges S., Tejada S., Pastor F., Villanueva H., Gallego J., Espinos J., Aristu J., Idoate M.A. (2012). Dendritic Cell Vaccination in Glioblastoma after Fluorescence-Guided Resection. World J. Clin. Oncol..

[20] Liau L.M., Ashkan K., Tran D.D., Campian J.L., Trusheim J.E., Cobbs C.S., Heth J.A., Salacz M., Taylor S., D’Andre S.D. (2018). First Results on Survival from a Large Phase 3 Clinical Trial of an Autologous Dendritic Cell Vaccine in Newly Diagnosed Glioblastoma. J. Transl. Med..

[21] Preusser M., van den Bent M.J. (2022). Autologous Tumor Lysate-Loaded Dendritic Cell Vaccination (DCVax-L) in Glioblastoma: Breakthrough or Fata Morgana?. Neuro Oncol..

[22] Cho D.-Y., Yang W.-K., Lee H.-C., Hsu D.-M., Lin H.-L., Lin S.-Z., Chen C.-C., Harn H.-J., Liu C.-L., Lee W.-Y. (2012). Adjuvant Immunotherapy with Whole-Cell Lysate Dendritic Cells Vaccine for Glioblastoma Multiforme: A Phase II Clinical Trial. World Neurosurg..

[23] Jie X., Hua L., Jiang W., Feng F., Feng G., Hua Z. (2012). Clinical Application of a Dendritic Cell Vaccine Raised Against Heat-Shocked Glioblastoma. Cell Biochem. Biophys..

[24] Yao L., Hatami M., Ma W., Skutella T. (2024). Vaccine-Based Immunotherapy and Related Preclinical Models for Glioma. Trends Mol. Med..

[25] Wen P.Y., Reardon D.A., Armstrong T.S., Phuphanich S., Aiken R.D., Landolfi J.C., Curry W.T., Zhu J.-J., Glantz M., Peereboom D.M. (2019). A Randomized Double-Blind Placebo-Controlled Phase II Trial of Dendritic Cell Vaccine ICT-107 in Newly Diagnosed Patients with Glioblastoma. Clin. Cancer Res..

[26] Ridolfi L., Gurrieri L., Riva N., Bulgarelli J., De Rosa F., Guidoboni M., Fausti V., Ranallo N., Calpona S., Tazzari M. (2024). First Step Results from a Phase II Study of a Dendritic Cell Vaccine in Glioblastoma Patients (CombiG-Vax). Front. Immunol..

[27] Lepski G., Bergami-Santos P.C., Pinho M.P., Chauca-Torres N.E., Evangelista G.C.M., Teixeira S.F., Flatow E., de Oliveira J.V., Fogolin C., Peres N. (2023). Adjuvant Vaccination with Allogenic Dendritic Cells Significantly Prolongs Overall Survival in High-Grade Gliomas: Results of a Phase II Trial. Cancers.

[28] Pasqualetti F., Zanotti S. (2023). Nonrandomised Controlled Trial in Recurrent Glioblastoma Patients: The Promise of Autologous Tumour Lysate-Loaded Dendritic Cell Vaccination. Br. J. Cancer.

[29] Wang X. (2015). A Systemic Review of Clinical Trials on Dendritic-Cells Based Vaccine Against Malignant Glioma. J. Carcinog. Mutagen..

[30] Tesileanu C.M.S., Sanson M., Wick W., Brandes A.A., Clement P.M., Erridge S.C., Vogelbaum M.A., Nowak A.K., Baurain J.-F., Mason W.P. (2022). Temozolomide and Radiotherapy versus Radiotherapy Alone in Patients with Glioblastoma, IDH-Wildtype: Post-Hoc Analysis of the EORTC Randomized Phase 3 CATNON Trial. Clin. Cancer Res..

